# A publicly accessible global data repository – the WHO TB-IPD platform

**DOI:** 10.5588/ijtldopen.24.0131

**Published:** 2024-04-01

**Authors:** R.L. Goodall, S.M. Fabiane, A. Hakiman, A.M. Crook, F. Mirzayev, S. Schumacher, M.X. Rangaka

**Affiliations:** ^1^ Medical Research Council Clinical Trials Unit at University College London (UCL), London, UK;; ^2^ World Health Organization, Geneva, Switzerland;; ^3^ Institute for Global Health, UCL, London, UK

**Keywords:** tuberculosis, meta-analysis, data sharing, treatment

TB treatment is complex and changing rapidly. Individual research studies provide information on efficacy and safety, but few are large and definitive.^1^ By pooling data, we obtain better evidence, especially if the data are consolidated at the level of the individual, rather than summary or aggregated data, as we increase statistical power to study hypotheses. In the past, the WHO have pooled data specifically for analyses that inform their Guideline Development Group (GDG).^2‒4^ In 2021, the WHO decided to support a publicly accessible global platform for individual patient data (IPD) on TB that would combine single datasets into a pooled resource to inform future guidelines and promote open science through onward sharing.^5^ Here we describe the governance, functionality and future direction of this platform, the TB-IPD.

**Figure 1.**
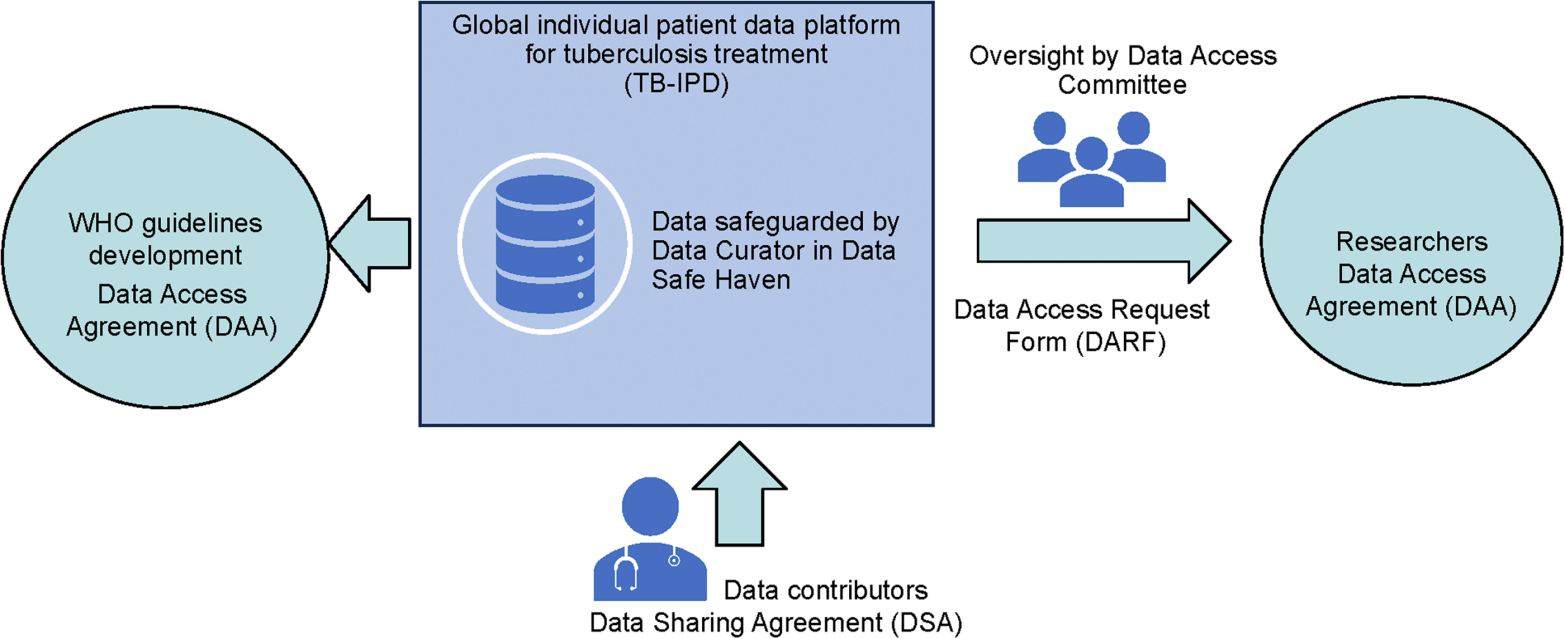
The TB-IPD Platform. IPD = individual patient data.

The purpose of the TB-IPD is to facilitate pooling of individual patient data provided by researchers or local/national TB programmes to expand the knowledge and understanding of TB globally, including informing future treatment guidelines. Although the initial concept note proposed an IPD that focused on drug-resistant TB and observational data, the remit of TB-IPD has been expanded to include trials of TB treatment (drug-susceptible, as well as drug-resistant TB). In addition, paediatric and pregnancy data are also included through a collaboration with Stellenbosch University, Cape Town, South Africa. University College London (UCL; London, UK) was selected as the data curator for TB-IPD, and the work therefore falls within the framework of research governance policies and procedures adopted by UCL to ensure research is carried out legally, ethically and with integrity. This includes legal agreements, secure handling and storage of data and procedures for review and oversight. A Data Access Committee, made up of data contributors, community representatives, the WHO and UCL (as a non-voting member), is responsible for reviewing requests made for data access. A steering committee is responsible for oversight and coordination of the project on a day-to-day basis, and to date has been focusing on setting up the TB-IPD platform. Figure 1 shows how TB-IPD works; data contributors share their effectively anonymised data with the data curator (UCL), and the data are stored in the Data Safe Haven, a technical solution certified to ISO 27001 information security standards.^6^ When required, the WHO requests that the data be provided to their data analyst for guideline development. Other researchers may also make requests for data to the Data Access Committee. The data held in TB-IPD are ‘effectively anonymised’, as defined by the Information Commissioner’s Office of the UK Government,^7,8^ which means that the data contain no personal identifiers and have a remote risk of being re-identified. The Data Safe Haven is UCL’s technical solution for storing, handling and analysing identifiable data. It is effectively a ‘walled garden’ with all storage and processing of data in a controlled environment with no access to the outside. Although the data are not identifiable, we use the Data Safe Haven for the TB-IPD as it provides extra security. Each data contributor is given a one-time time-limited access to upload their data, which is then checked for malware, before being released to the Data Safe Haven. All data fields are checked to ensure that no personal identifiers have been included in error, and the data incorporated into TB-IPD. When data are released to researchers, the data are effectively anonymised by ensuring that there are no personal identifiers (date of birth is replaced by year of birth or age at treatment start, country is replaced by WHO region), and dates may be shifted by a random number of days or converted to number of days from start of treatment. In addition, only a minimal dataset of variables required for the specified analysis will be shared with researchers in accordance with the principles of data minimisation.^9^

**Figure 2.**
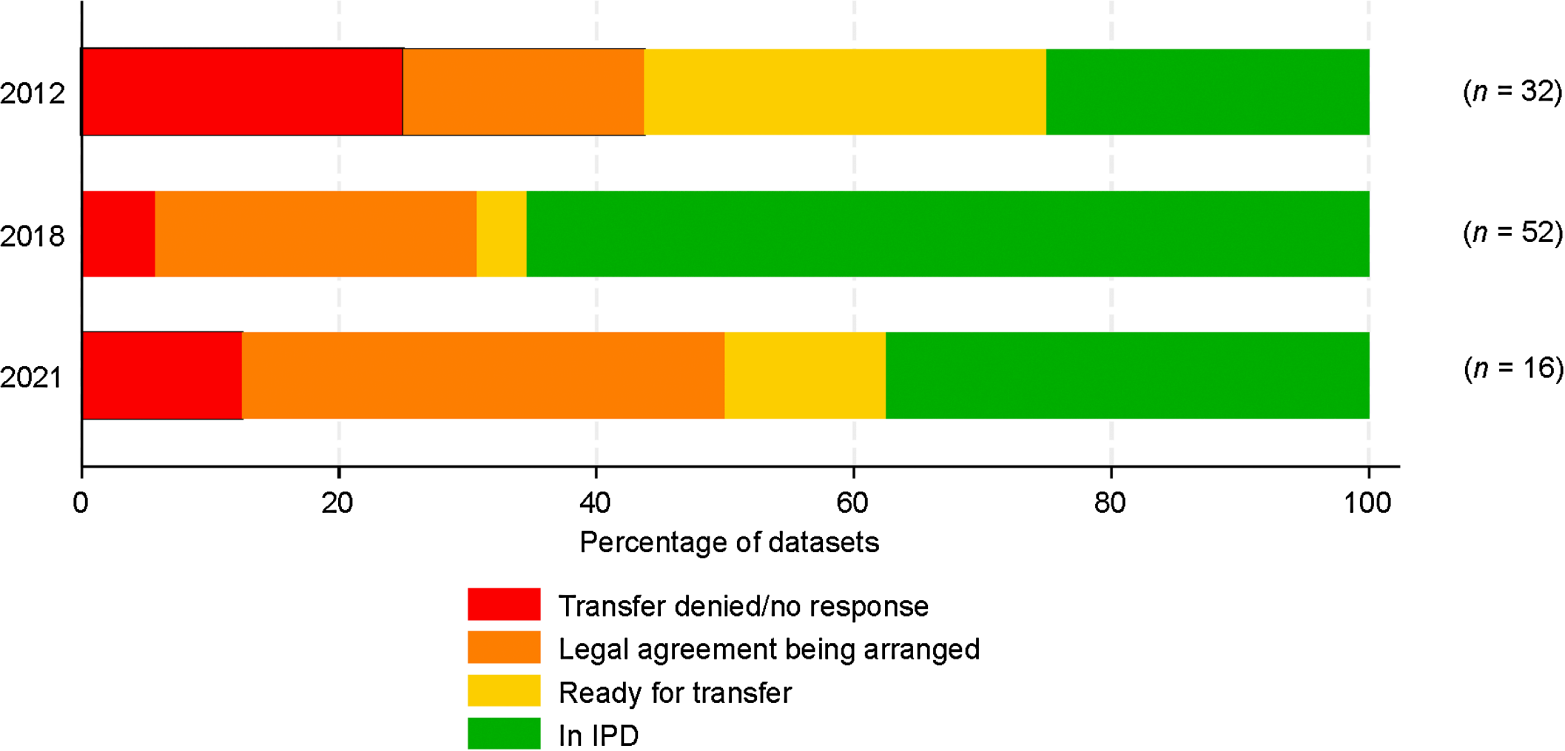
Status of historical datasets in TB-IPD Platform. IPD = individual patient data.

Figure 1 also shows how three important documents, the Data Sharing Agreement, the Data Access Request Form and Data Access Agreement, enable the process. Collectively, these documents cover legal requirements for data protection, mutual liability, non-commercial use, protection of intellectual property and recognition within publications. They also describe requests for data, including a statistical analysis plan. These documents are available on the TB-IPD website,^10^ along with the Data Dictionary, which specifies the format of observational data held within the platform. The Data Dictionary was originally based on requirements for the GDG but has been revised and updated for future datasets contributed to the IPD platform.^11^ Data include demographics, characteristics at the time of starting treatment, microbiology, treatment outcomes and safety events. Clinical trials are received in the format of the trial database, along with the protocol and case report forms. The TB-IPD currently holds information on 35,981 individuals from 49 datasets. This includes individual-level data collated for the previous GDG held at McGill University, Montreal, QC, Canada (2012, 2018) and the University of Sydney, Sydney, NSW, Australia (2021). For each dataset, the data owners have been contacted and permission sought for inclusion within TB-IPD. At present, 48 out of 100 (48%) historical datasets have been transferred to the new platform, 14 are about to be transferred, and for 25 we are in discussions to finalise the legal documentation for transfer (see Figure 2).

Looking ahead, there will be calls for data for future GDG meetings, but dataset owners may also approach members of the Steering Committee to discuss adding their data to TB-IPD. Data shared for GDG analyses may carry an embargo on forward sharing to other researchers. Although this is not aligned with the ethos of the project, it is possible to put a time-limited embargo on data while primary researchers complete their analyses before granting wider access to the community. Future plans include broadening access to existing datasets with sponsorship, supported access and analyses driven by the Global South through annual awards of seed-funding. We aim to increase the recognition of the datasets that contributed to the platform by assigning digital object identifiers (doi's) to each, and indexing by Web of Science. User experience will also be enhanced through in-built web-based interactive analytics, enabling improved data visualisation. We believe that a global data repository will help drive the research agenda, allowing us to make evidence-based decisions for more effective TB treatment and patient care.
